# Protein Concentrations of Thrombospondin-1, MIP-1β, and S100A8 Suggest the Reflection of a Pregnancy Clock in Mid-Trimester Amniotic Fluid

**DOI:** 10.1007/s43032-020-00229-z

**Published:** 2020-10-07

**Authors:** Felicia Viklund, Maria Hallingström, Marian Kacerovsky, Teresa Cobo, Kristin Skogstrand, David M. Hougaard, Karin Sävman, Ylva Carlsson, Panagiotis Tsiartas, Julius Juodakis, Staffan Nilsson, Bo Jacobsson

**Affiliations:** 1 Stockholm South General Hospital, Stockholm, Sweden; 2 Department of Obstetrics and Gynecology, Sahlgrenska University Hospital/Östra, Gothenburg, Sweden; 3 Department of Obstetrics and Gynecology, Institute of Clinical Sciences, Sahlgrenska Academy, University of Gothenburg, Gothenburg, Sweden; 4 Biomedical Research Center, University Hospital Hradec Kralove, Hradec Kralove, Czech Republic; 5 Department of Obstetrics and Gynecology, University Hospital Hradec Kralove, Charles University, Faculty of Medicine in Hradec Kralove, Hradec Kralove, Czech Republic; 6 BCNatal - Barcelona Center for Maternal-Fetal and Neonatal Medicine (Hospital Clínic and Hospital Sant Joan de Deu), Fetal i+D Fetal Medicine Research Center, Institut d’Investigacions Biomèdiques Agustí Pi I Sunyer (IDIBAPS), University of Barcelona, Barcelona, Spain; 7 Centro de Investigaciones Biomedicas en Enfermedades Raras (CIBER-ER), Barcelona, Spain; 8 Center for Neonatal Screening, Department of Congenital Disorders, Statens Serum Institut, Copenhagen, Denmark; 9 Department of Pediatrics, Institute of Clinical Sciences, Sahlgrenska Academy, University of Gothenburg, Gothenburg, Sweden; 10 Department of Neonatology, The Queen Silvia Children’s Hospital, Sahlgrenska University Hospital, Gothenburg, Sweden; 11 Present Address: School of Maths and Stats, Victoria University of Wellington, Wellington, New Zealand; 12 Department of Mathematical Sciences, Chalmers University of Technology, Gothenburg, Sweden; 13 Department of Pathology and Genetics, Institute of Biomedicine, Sahlgrenska Academy, University of Gothenburg, Gothenburg, Sweden; 14 Department of Genetics and Bioinformatics, Division of Health Data and Digitalisation, Institute of Public Health, Oslo, Norway

**Keywords:** Amniotic fluid, Gestational duration, Inflammation, Mid-trimester, Proteins

## Abstract

The development of immunoassays enables more sophisticated studies of the associations between protein concentrations and pregnancy outcomes, allowing early biomarker identification that can improve neonatal outcomes. The aim of this study was to explore associations between selected mid-trimester amniotic fluid proteins and (1) overall gestational duration and (2) spontaneous preterm delivery. A prospective cohort study, including women undergoing mid-trimester transabdominal genetic amniocentesis, was performed in Gothenburg, Sweden, 2008–2016 (*n* = 1072). A panel of 27 proteins related to inflammation was analyzed using Meso-Scale multiplex technology. Concentrations were adjusted for gestational age at sampling, experimental factors, year of sampling, and covariates (maternal age at sampling, parity (nulliparous/multiparous), smoking at first prenatal visit, and in vitro fertilization). Cox regression analysis of the entire cohort was performed to explore possible associations between protein concentrations and gestational duration. This was followed by Cox regression analysis censored at 259 days or longer, to investigate whether associations were detectable in women with spontaneous preterm delivery (*n* = 47). Finally, linear regression models were performed to analyze associations between protein concentrations and gestational duration in women with spontaneous onset of labor at term (*n* = 784). HMG-1, IGFBP-1, IL-18, MIP-1α, MIP-1β, S100A8, and thrombospondin-1 were significantly associated with gestational duration at term, but not preterm. Increased concentrations of thrombospondin-1, MIP-1β, and S100A8, respectively, were significantly associated with decreased gestational duration after the Holm-Bonferroni correction in women with spontaneous onset of labor at term. This adds to the concept of a pregnancy clock, where our findings suggest that such a clock is also reflected in the amniotic fluid at early mid-trimester, but further research is needed to confirm this.

## Introduction

Gestational duration has a significant impact on the short- and long-term health of the neonate [1–3]. Data indicate that an inflammatory process precedes the onset of labor in both term and preterm pregnancies [4–6] and that endocrine, mechanical, and genetic factors are involved [7–9]. However, the mechanisms of pregnancy maintenance and timing of delivery are not yet fully understood [10]. The concept of a pregnancy clock, in which chronological and coordinated signals from the fetus, fetal membranes, placenta, decidua, and myometrium modulate the duration of pregnancy [11–13], has recently been expanded with the concepts of a proteomic clock and an immune clock [14, 15]. It has been suggested that recognition of disruptive patterns in these chronological signals could enable early detection of pregnancy complications [15].

Amniotic fluid is a biological surrogate for the dynamic environment surrounding the developing fetus, and its origin and composition changes as pregnancy progresses [16]. Previous studies have identified inflammatory biomarkers that are associated with gestational duration in the amniotic fluid at mid-trimester [17, 18]. However, these studies have mainly focused on spontaneous preterm delivery (PTD, < 37 + 0 gestational weeks) and have also been limited to one or a few proteins. The statistical power to detect associations increases when gestational duration is considered to be a continuous variable instead of a dichotomous (term/preterm) trait [19]. A continuous gestational duration variable is also clinically relevant as there is a gradient of increasing risk of adverse neonatal outcome with decreasing gestational duration [1, 20]. Furthermore, the development of multiplex immunoassays, which allow simultaneous analysis of multiple analytes from small sample volumes, has enabled a more explorative approach to protein patterns in biological compartments in relation to pregnancy outcome [21].

This study aimed at evaluating the concentrations of 27 selected proteins in the amniotic fluid of asymptomatic women at mid-trimester in relation to gestational duration and spontaneous PTD, using multiplex technology. The assay panel selected was designed to explore the inflammatory process that precedes the onset of labor.

## Methods

### Study Design and Participants

In this prospective cohort study, pregnant women were recruited at Sahlgrenska University Hospital/Östra, Gothenburg, Sweden, between September 2008 and June 2016. The women were enrolled before the clinical introduction of noninvasive prenatal testing (NIPT) in 2017. Inclusion criteria were maternal age ≥ 18 years with a viable singleton pregnancy and undergoing genetic amniocentesis at 14–19 gestational weeks. Clinical indications for genetic amniocentesis were maternal age ≥ 35 years, high risk found in the first-trimester combined screening, anxiety, or family history of a chromosomal abnormality or genetic disease. Multiple pregnancy, positive for HIV or hepatitis B, and known or suspected fetal malformations were ineligibility criteria, as were situations in which study samples could not be collected. Women were excluded if they declined participation, could not give informed consent in Swedish due to language difficulties or if an insufficient amount of fluid was retrieved during amniocentesis.

Demographics and pregnancy outcomes were obtained by review of medical records. Gestational age was based on fetal biometry at ultrasound, routinely performed at gestational weeks 17–20. Spontaneous PTD was defined as delivery < 37 gestational weeks, as a result of either preterm labor or preterm prelabor rupture of membranes. Women with medically indicated (iatrogenic) PTD, where various complications affected the duration of pregnancy, as well as women who had a miscarriage, stillbirth, termination of pregnancy, incomplete data or who were lost to follow-up, were excluded from analysis.

### Sample Collection and Processing

Amniocentesis was performed transabdominally with a 22-gauge needle under sonographic guidance. An additional 3 ml of fluid was aspirated for research purposes. Samples were stored at + 4–8 °C immediately after sampling and coded. Samples were centrifuged for 20 min at 12,000 *g* at 4 °C to separate supernatant from the pellet. Aliquots were stored at − 80 °C until analysis. None of the aliquots used in this study had been thawed or used in previous analyses.

### Development of the Assay Panel

The assay panel was designed to explore the inflammatory processes and mechanisms preceding the onset of labor, both at term and at preterm. Selection was influenced by previous studies reporting biomarkers associated with term and preterm parturition [6, 22] as well as proteins previously analyzed in a sub-cohort of this project [23]. The selection of DAMPs in the panel was based on the hypothesis that these endogenous mediators trigger an inflammatory process, defined as sterile intra-amniotic inflammation [24, 25], related to the onset of labor both at term [26] and preterm [27].

The panel consisted of 27 cytokines, chemokines, damage-associated molecular patterns (DAMPs), and other proteins, distributed on two 10-plex analysis for analytes that had previously been developed and tested by the institute performing the analyses (panels 1 and 2) and one 7-plex analysis with analytes that had not previously been tested (panel 3).

### Analysis of Samples

Analyte concentrations were analyzed at Statens Serum Institut (SSI), Copenhagen, Denmark, using in-house multiplex sandwich immunoassays based on U-PLEX Meso-Scale technology. Samples from spontaneous PTD cases were evenly distributed on the plates by the researchers, to minimize the risk of confounding by the plate layout. Laboratory staff performing the analyses were blinded to clinical information and outcome.

The analysis started with the different capture antibodies being biotinylated, after which they were bound to different linkers (1–10), mixed to reach a concentration of 10 μg/ml per antibody, added to each of the U-PLEX plate (Meso-Scale, K15235) wells (50 μl/well) in all plates in the respective panels and incubated for 1 h. After washing with washing buffer (PBS, containing 0.05% Tween 20), the plates were stored at 4 °C until use. Detection antibodies were sulfo-tagged using Meso-Scale Discovery Gold Sulfo-Tag NHS-Ester (Meso-Scale, R91AO-2). Monocyte chemotactic protein 1 (C-C motif chemokine 2; MCP-1) antibody was purchased from BD Biosciences, while the remaining antibodies came from R&D Systems. Calibrators, consisting of eight samples with known concentrations, were used to create calibration curves to translate readings into concentrations. High and low controls were used to calculate assay variations. Both calibrators and high and low controls were prepared by recombinant antigens and pipetted onto each plate, together with 25μl of samples. The amniotic fluid samples were measured undiluted in panel 1 and panel 3 and diluted 1:5 in panel 2. The plates were sealed, simultaneously incubated and shaken for 2 h and washed three times. The plates were then simultaneously incubated and shaken for another 2 h after adding the corresponding detection antibodies. The plates were washed and 150 μl 2xRead buffer T (Meso-Scale R92TC) per well was added. They were then immediately read on the QuickPlex reader.

### Statistical Analyses

Continuous data were presented using median and interquartile range (IQR), while categorical data were presented as frequency distribution. Concentrations were log-transformed for statistical analysis. Amniotic fluid protein concentrations were adjusted for gestational age at sampling, experimental factors (plates), and year of sampling using linear regression. In the following analysis, these adjusted concentrations were used as predictors, together with covariates selected based on previous studies (maternal age at sampling, parity (nulliparous/multiparous), smoking at first prenatal visit, and in vitro fertilization (IVF)) [28–31]. For each of the amniotic fluid protein concentrations, separately, a Cox regression analysis of the entire study cohort was used to explore possible associations with gestational duration. Cox regression was then censored at 259 days (37 + 0 gestational weeks) or longer to examine whether associations could be detected in women with spontaneous PTD. Linear regression models, followed by a conservative Holm-Bonferroni correction, were used to evaluate protein concentrations’ associations with gestational duration as a continuous variable in women with spontaneous onset of labor at term, as well as to obtain an effect estimate of days/standard deviation (SD). A *p* value of < 0.05 using a two-sided alternative hypothesis was considered significant. Statistical analysis was performed in SPSS 25.0 for Windows XP OS (SPSS Inc., USA) and R, version 3.3.1.

## Results

### Characteristics of the Study Population

**Fig. 1:**
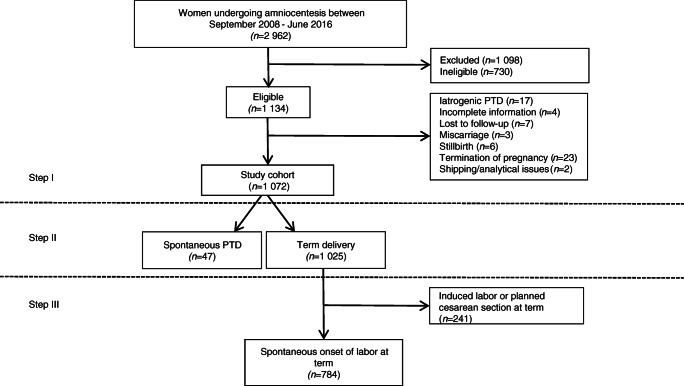
Flow chart. This figure shows the selection process for the different cohorts in the respective analytical steps. In step I, the entire cohort was analyzed. Step II consists of women with spontaneous PTD, compared with women with term delivery, and step III consists of women with spontaneous onset of labor at term

Between September 2008 and June 2016, 2962 women underwent mid-trimester genetic amniocentesis at the department. After application of the exclusion and ineligibility criteria described above, the study cohort consisted of 1072 women. The selection of study participants is displayed in Fig. 1. Maternal and neonatal characteristics are presented in Table 1.

**Table 1:** Maternal and neonatal characteristics

Variable	Study cohort (*n* = 1072)	Sub-analysis			Spontaneous onset of labor at term (*n* = 784)
		Spontaneous preterm delivery (*n* = 47)	Term delivery (*n* = 1025)	***p***	
Maternal age at sampling (years)	37 (34–39)	37 (35–39)	37 (34–39)	0.895	37 (34–39)
Nulliparous	294 (27.4%)	19 (40.4%)	275 (26.8%)	**0.046**	210 (26.8%)
Maternal BMI at first prenatal visit	23.5 (21.5–26.0)	25.1 (22.8–27.1)	23.4 (21.5–26.0)	**0.024**	23.4 (21.5–26.0)
Smoking at first prenatal visit	54 (5.0%)	5 (10.6%)	49 (4.8%)	0.082	32 (4.1%)
IVF	35 (3.3%)	6 (12.8%)	29 (2.8%)	**0.003**	16 (2.0%)
Previous preterm delivery	75 (7.0%)	7 (14.9%)	68 (6.6%)	**0.040**	48 (6.1%)
Gestational age at sampling (weeks + days)	15 + 5 (15 + 3–16 + 1)	15 + 4 (15 + 1–16 + 0)	15 + 5 (15 + 3–16 + 1)	0.062	15 + 5 (15 + 2–16 + 1)
Gestational duration (weeks + days)	39 + 6 (38 + 5–40 + 6)	35 + 6 (33 + 4–36 + 4)	39 + 6 (38 + 6–40 + 6)	**< 0.001**	40 + 0 (39 + 1–40 + 6)
Mode of delivery					
Vaginal	762 (71.1%)	34 (72.3%)	728 (71.0%)	1.000	656 (83.7%)
Vacuum extraction/forceps	59 (5.6%)	1 (2.1%)	58 (5.7%)	0.511	50 (6.4%)
Cesarean section	251 (23.4%)	12 (25.5%)	239 (23.3%)	0.726	78 (9.9%)
Birth weight (grams)	3540 (3231–3885)	2645 (2240–2910)	3560 (3275–3900)	**< 0.001**	3575 (3280–3905)
Gender					
Male	544 (50.7%)	21 (44.7%)	523 (51.0%)	0.456	401 (51.1%)
Female	528 (49.3%)	26 (55.3%)	502 (49.0%)		383 (48.9%)
Apgar score < 7 at 5 min	14 (1.3%)	1 (2.1%)	13 (1.3%)	0.468	12 (1.5%)

This table presents the maternal and neonatal characteristics of the final study population, a comparison between women with spontaneous preterm delivery and term delivery, as well as the group with spontaneous onset of labor at term. Continuous variables were analyzed using the nonparametric Mann-Whitney *U* test and presented as median (IQR). Categorical variables were analyzed using Fisher’s exact test and presented as number (%). Bold values indicates statistical significance at *p* < 0.05 using a two-sided alternative hypothesis

### Candidate Protein Concentrations

The inter-assay coefficients of variation (CV), intra-assay CV, and limits of detection (LOD) for the analytes measured are presented in Table 2. The protein names and, in some cases, the recommended names (in parentheses) and their short-form abbreviations are assigned by the UniProt Consortium [32]. In cases where the protein concentrations reported from SSI were too low or too high to be fitted on the standard curve, values were set at the LOD or at the highest concentration that could be measured for that specific analyte. Triggering receptor expressed on myeloid cells 1 (TREM-1) was one of the initial proteins in panel 2. However, due to cross-reactions in the preparation phase, this protein was not analyzed. A total of 26 analytes were thus measured, and panel 2 consisted of a 9-plex analysis instead. The median concentrations of the 26 examined proteins are presented in Table 3. The majority of the protein concentrations were not affected by storage time, with the exception of IL-10, TNF-β, CRP, MIP-1α, RANTES, and MMP-8.

**Table 2:** Analytical variability and limit of detection

Protein name	Short protein name	Inter-assay CV	Intra-assay CV	LOD	Samples below the LOD
Adiponectin	Adiponectin	22.6	4.4	0.006 ng/ml	0
Brain-derived neurotrophic factor	BDNF	24.7	15.0	31.4 pg/ml	0
C-reactive protein	CRP	37.1	8.0	0.0002 μg/ml	0
Granulocyte-macrophage colony-stimulating factor	GM-CSF	30.3	15.5	53.0 pg/ml	0
High mobility group protein B1	HMG-1	25.4	7.2	5.67 ng/ml	0
Heat shock protein 70	HSP70	35.9	29.3	0.44 ng/ml	0
Insulin-like growth factor-binding protein 1	IGFBP-1	15.5	3.9	0.035 ng/ml	0
Interleukin-1 beta	IL-1β	20.6	8.9	0.26 pg/ml	0
Interleukin-6	IL-6	24.1	4.6	0.99 pg/ml	0
Interleukin-8	IL-8	9.4	9.1	0.25 pg/ml	0
Interleukin-10	IL-10	34.2	13.4	0.25 pg/ml	0
Interleukin-12	IL-12	39.4	11.7	0.57 pg/ml	0
Interleukin-17	IL-17	22.4	11.6	1.47 pg/ml	0
Interleukin-18	IL-18	21.5	9.2	2.54 pg/ml	0
Monocyte chemotactic protein 1 (C-C motif chemokine 2)	MCP-1	36.4	12.2	3.84 pg/ml	0
Macrophage inflammatory protein-1 alpha (C-C motif chemokine 3)	MIP-1α	19.3	4.8	1.2 pg/ml	0
Macrophage inflammatory protein-1 beta (C-C motif chemokine 4)	MIP-1β	37.6	3.2	25.7 pg/ml	5
Matrix metalloproteinase-8 (neutrophil collagenase)	MMP-8	33.4	2.7	0.005 ng/ml	0
Matrix metalloproteinase-9	MMP-9	26.5	5.8	0.004 ng/ml	1
T cell-specific protein RANTES (C-C motif chemokine 5)	RANTES	26.1	9.4	0.4 pg/ml	19
S100 calcium-binding protein A8	S100A8 (protein S100-A8)	33.4	5.5	10.7 ng/ml	1
Transforming growth factor beta-1	TGF-β1	44.6	5.1	2.03 ng/ml	0
Tumor necrosis factor alpha	TNF-α	17.6	5.6	8.4 pg/ml	0
Tumor necrosis factor beta (lymphotoxin-alpha)	TNF-β/LT-α	14.3	10.2	0.26 pg/ml	0
Soluble tumor necrosis factor receptor-1	sTNF-RI	13.7	9.8	9.36 pg/ml	0
Thrombospondin-1	Thrombospondin-1	27.6	3.5	0.85 ng/ml	0

This table presents inter-assay coefficient of variation (CV), intra-assay CV, limit of detection (LOD), and the number of samples below the LOD for the measured proteins

**Table 3:** Protein concentrations

Short protein name	Protein concentration	Unit	Panel number
Adiponectin	11.98 (7.17–20.00)	ng/ml	2
BDNF	1061.10 (767.40–1377.32)	pg/ml	2
CRP	29.82 (18.03–35.24)	μg/ml	2
GM-CSF	334.02 (257.27–449.52)	pg/ml	1
HMG-1	380.26 (224.51–648.23)	ng/ml	3
HSP70	78.38 (52.26–125.00)	ng/ml	2
IGFBP-1	407.27 (364.70–448.01)	ng/ml	2
IL-1β	2.00 (1.32–3.03)	pg/ml	1
IL-6	708.33 (327.32–1605.70)	pg/ml	3
IL-8	864.64 (336.23–2199.61)	pg/ml	1
IL-10	2.32 (1.72–3.32)	pg/ml	1
IL-12	34.04 (24.18–48.40)	pg/ml	1
IL-17	46.17 (33.44–68.21)	pg/ml	1
IL-18	21.51 (16.98–27.24)	pg/ml	1
MCP-1	772.08 (561.14–1113.44)	pg/ml	1
MIP-1α	365.38 (231.78–548.24)	pg/ml	2
MIP-1β	204.13 (139.70–352.40)	pg/ml	3
MMP-8	6.25 (2.83–15.92)	ng/ml	3
MMP-9	3.82 (2.53–6.12)	ng/ml	2
RANTES	87.36 (74.37–103.31)	pg/ml	2
S100A8	177.88 (112.98–280.03)	ng/ml	3
TGF-β1	168.45 (106.60–242.21)	ng/ml	2
TNF-α	301.35 (194.78–468.20)	pg/ml	3
TNF-β	2.50 (1.78–3.46)	pg/ml	1
sTNF-RI	12.45 (10.72–14.45)	pg/ml	1
Thrombospondin-1	329.90 (185.14–552.04)	ng/ml	3

Protein concentrations were calculated from raw data and are presented as median (IQR). The respective unit of each analyte and the panel in which the analyte was included are also shown in the table

### Protein Concentrations and Gestational Age at Sampling

Seventeen of the 26 proteins were significantly associated with gestational age at sampling (Table 4). Thrombospondin-1 and HMG-1 underwent the most substantial concentration changes, a 3.4% increase and a 3.2% increase in concentration per day, respectively.

**Table 4:** Relationship between protein concentrations and gestational age at sampling

Short protein name	Percentage change per day	*p*
Adiponectin	1.17	**0.007**
BDNF	− 0.74	**0.001**
CRP	− 1.97	**2E-06**
GM-CSF	− 0.12	0.43
HMG-1	3.15	**1E-10**
HSP70	− 0.37	0.12
IGFBP-1	0.18	**0.011**
IL-1β	− 0.30	0.24
IL-6	− 2.01	**0.002**
IL-8	− 2.78	**0.0003**
IL-10	0.19	0.38
IL-12	− 0.33	0.15
IL-17	− 0.02	0.95
IL-18	1.60	**3E-15**
MCP-1	1.31	**3E-06**
MIP-1α	− 1.00	**0.002**
MIP-1β	1.60	**5E-09**
MMP-8	− 2.45	**0.001**
MMP-9	2.05	**2E-06**
RANTES	− 0.28	0.49
S100A8	2.67	**9E-10**
TGF-β1	− 0.99	**0.002**
TNF-α	2.40	**6E-10**
TNF-β	− 0.19	0.41
sTNF-RI	0.14	0.22
Thrombospondin-1	3.44	**4E-15**

This table presents the associations between protein concentrations in mid-trimester amniotic fluid and gestational age at sampling in the study cohort (*n* = 1072). The right-hand column presents the percentage change per day that sampling was postponed, where negative values represent a decreased concentration and positive values represent an increase. Bold text indicates statistical significance at *p* < 0.05 using a two-sided alternative hypothesis. Log protein concentrations were regressed on gestational age at sampling, adjusting for experimental factors (plates) and year of sampling

### Protein Concentrations and Gestational Duration

#### Step I: The Entire Study Cohort (*n* = 1072)

The median gestational duration in this cohort was 39 + 6 (IQR, 38 + 5–40 + 6) weeks. HMG-1, IGFBP-1, IL-18, MIP-1α, MIP-1β, S100A8, and thrombospondin-1 concentrations were significantly associated with gestational duration in the Cox regression analysis (Table 5).

**Table 5:** Associations between protein concentrations and gestational duration

Study cohort (*n* = 1072)			Spontaneous onset of labor at term (*n* = 784)		
Short protein name	HR (SD)	*p*	Days per SD	*p*	FDR (*q*)
Thrombospondin-1	1.10	**0.009**	− 1.1	0.001*	0.003
MIP-1β	1.11	**0.002**	− 0.9	< 0.001*	0.008
S100A8	1.08	**0.020**	− 0.9	0.002*	0.013
IL-18	1.09	**0.011**	− 0.8	**0.004**	0.027
MIP-1α	1.08	**0.020**	− 0.7	**0.015**	0.06
IGFBP-1	1.12	**0.002**	− 0.7	**0.010**	0.045
HMG-1	1.08	**0.023**	− 0.7	**0.010**	0.045
TNF-α	1.06	0.09	− 0.6	**0.031**	0.10
Adiponectin	1.02	0.54	− 0.5	0.09	0.25
sTNF-RI	1.06	0.10	− 0.4	0.10	0.25
CRP	1.04	0.30	− 0.4	0.18	0.34
MMP-9	1.05	0.17	− 0.4	0.16	0.34
RANTES	1.03	0.34	− 0.4	0.10	0.25
TGF-β1	1.05	0.16	− 0.4	0.18	0.34
MCP-1	1.04	0.24	− 0.3	0.27	0.45
GM-CSF	0.98	0.58	0.3	0.25	0.43
BDNF	1.04	0.26	− 0.3	0.32	0.49
IL-1β	0.99	0.73	0.2	0.53	0.71
IL-12	1.00	0.95	− 0.2	0.58	0.72
IL-10	1.00	0.98	− 0.1	0.67	0.76
MMP-8	0.97	0.47	0.2	0.47	0.68
IL-6	0.99	0.77	0.2	0.55	0.71
TNF-β	1.01	0.86	− 0.1	0.63	0.74
HSP70	1.02	0.48	− 0.1	0.78	0.85
IL-8	1.00	0.97	− 0.04	0.89	0.89
IL-17	0.99	0.75	0.04	0.87	0.89

A Cox regression analysis was performed on the logarithmically transformed protein concentrations that were pre-adjusted for gestational age at sampling, experimental factors (plates), and year of sampling, using maternal age at sampling, parity, smoking at first prenatal visit, and IVF as covariates. The results are presented as hazard ratios (HR) per standard deviation (SD). A linear regression model was performed for the group with spontaneous onset of labor at term, and the results are presented as days per SD. The table is sorted by *p* value for the group with spontaneous onset of labor at term. Bold text indicates nominal statistical significance at *p* < 0.05 using a two-sided alternative hypothesis. *p* values are additionally adjusted by false discovery rate (FDR) (*q* values) and by Holm-Bonferroni. Remaining significance after Holm-Bonferroni correction is indicated by an asterisk

#### Step II: Women with Spontaneous Preterm Delivery (*n* = 47)

The incidence of spontaneous PTD in the entire study cohort was 4.4% (47/1072). Women with spontaneous PTD were more often nulliparous, had a higher BMI at the first prenatal visit, and had a higher rate of IVF and previous PTD than women with a term delivery (Table 1).

The median gestational duration among women with spontaneous PTD was 35 + 6 (IQR, 33 + 4–36 + 4) weeks, compared with 39 + 6 (IQR, 38 + 6–40 + 6) weeks in women who underwent term delivery.

The selected proteins were not associated with spontaneous PTD in the censored Cox regression model (Table 6), indicating that the associations found in step I were mainly derived from women with term delivery. Women with spontaneous PTD were therefore excluded from further analysis.

**Table 6:** Association between protein concentrations and spontaneous preterm delivery

Spontaneous preterm delivery (*n* = 47)		
Short protein name	HR (SD)	*p*
Adiponectin	0.79	0.10
BDNF	1.00	0.98
CRP	1.08	0.63
GM-CSF	1.18	0.28
HMG-1	1.01	0.93
HSP70	0.94	0.64
IGFBP-1	0.85	0.21
IL-1β	1.06	0.70
IL-6	0.98	0.88
IL-8	0.98	0.87
IL-10	0.96	0.80
IL-12	0.95	0.70
IL-17	0.93	0.62
IL-18	1.17	0.27
MCP-1	1.24	0.10
MIP-1α	1.11	0.46
MIP-1β	0.98	0.90
MMP-8	0.77	0.10
MMP-9	1.08	0.60
RANTES	0.93	0.56
S100A8	0.90	0.46
TGF-β1	1.03	0.82
TNF-α	0.97	0.81
TNF-β	0.82	0.18
sTNF-RI	0.87	0.37
Thrombospondin-1	0.83	0.17

A Cox regression analysis, with 1025 deliveries censored at 37 weeks, was performed on the logarithmically transformed protein concentrations that were pre-adjusted for gestational age at sampling, experimental factors (plates), and year of sampling, using maternal age at sampling, parity, smoking at first prenatal visit, and IVF as covariates. The results are presented as hazard ratios (HR) per standard deviation (SD)

#### Step III: Women with Spontaneous Onset of Labor at Term (*n* = 784)

**Fig. 2:**
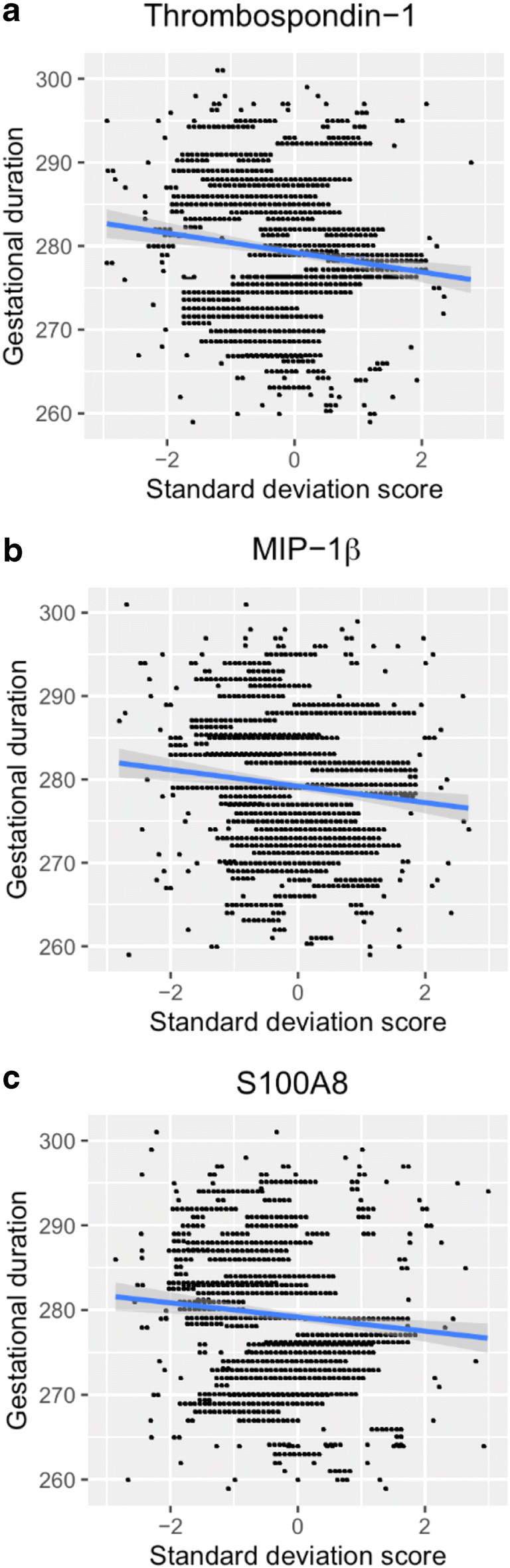
Scatter plots. The figure depicts the association between concentrations of **a** thrombospondin-1, **b** MIP-1β, and **c** S100A8 and gestational duration

Consequently, the subsequent analyses focused on women with term delivery. Women with induced labor or planned cesarean section at term (*n* = 241), for whom clinical decisions determined the gestational duration, were excluded from analysis, leaving only women with spontaneous onset of labor at term (*n* = 784). The median gestational duration in this cohort was 40 + 0 (IQR, 39 + 1–40 + 6) weeks. There were significant associations between thrombospondin-1, MIP-1β, S100A8, IL-18, MIP-1α, IGFBP-1, HMG-1, and TNF-α concentrations and gestational duration in this cohort (Table 5). All but TNF-α remained significant at a false discovery rate (FDR) of 0.1. Thrombospondin-1, MIP-1β, and S100A8 remained significant (*p*_c_ < 0.05) after a conservative Holm-Bonferroni correction. Scatter plots of the association between the concentrations of thrombospondin-1, MIP-1β, and S100A8 and gestational duration are presented in Fig. 2. For thrombospondin-1, an increase of one standard deviation (SD) in log concentration (corresponding to a 104% increase in concentration) was associated with a 1.1-day decrease in gestational duration. For MIP-1β and S100A8, an increase of one SD in log concentrations (corresponding to a 103% and 57% increase, respectively, in concentration) was associated with a shortened gestational duration by 0.9 day.

## Discussion

The key findings were as follows: (i) gestational age at sampling was significantly associated with concentrations of the majority of the selected analytes; (ii) mid-trimester amniotic fluid concentrations of thrombospondin-1, MIP-1β, and S100A8 were significantly associated with gestational duration in women with spontaneous onset of labor at term; and iii) mid-trimester amniotic fluid concentrations of the selected proteins were not associated with spontaneous PTD in this small cohort.

The concentrations of 17 of the 26 candidate proteins were associated with gestational age at sampling despite the limited sampling window of approximately 5 weeks. To our knowledge, this association has not been described to this extent previously, implying that the findings are an essential contribution to the research field and indicating the need to adjust for this variable.

In genetic studies, analyzing gestational duration has proven more successful than dichotomizing this trait, suggesting that a single biological pathway common to all preterm cases is unlikely to be identified [9, 33]. Several of the examined proteins in our study were associated with gestational duration, but these associations appeared to be driven by the term delivery group rather than the spontaneous PTD group. These findings are unique as only a few studies have examined amniotic fluid protein concentrations with gestational duration as a continuous variable [17, 18]. These studies have found an inverse relationship between IL-6 and IL-10 concentrations and gestational duration using enzyme-linked immunosorbent assay (ELISA), a singleplex assay. Their cohorts were small, with a spontaneous PTD rate of 7.2–15.6% and a substantially higher proportion of early PTD. Different assays, cohort sizes, and differences within study populations, such as maternal age, ethnicity, or PTD etiologies, may explain the contradictory results. Further, the risk of PTD in Sweden is generally low [34]. Even in this high-risk cohort, only 4.4% had a spontaneous PTD, limiting the amount of cases to be studied.

Identifying markers specific to spontaneous PTD has proven difficult. Several previous studies, including two by our group [23, 35], have investigated whether mid-trimester amniotic fluid proteins are associated with subsequent spontaneous PTD. The results are conflicting, as some have found associations [36–39], while others have not [23, 35, 40–42]. In this study, none of the selected proteins were associated with spontaneous PTD. However, spontaneous PTD cases of this study mainly occurred late, and infection and inflammation were thus involved to a lesser extent [43]. Our findings support spontaneous PTD as a multifactorial condition with different sub-phenotypes, making early identification of genetic or protein markers more challenging and complex. Furthermore, spontaneous PTD can also originate as a result of acute events which may not be detectable in amniotic fluid as early as at mid-trimester.

Similar issues have been encountered in genetic studies of gestational duration. Several genes associated with this trait have been identified [9, 33, 44], and many of them appear related to inflammatory processes. However, large sample sizes were required to achieve this, and most of the “hits” are still not consistent across cohorts.

Inflammation of non-infectious origin—or sterile inflammation—mediated by DAMPs or alarmins [27], has gained increasing attention. DAMPs are endogenous molecules released in response to cellular injury and death, eliciting an inflammatory response to defend the host through pathways ultimately leading to the release of pro-inflammatory cytokines [45]. This sterile inflammation has previously been described in relation to several pregnancy complications [46], such as spontaneous PTD [27], but also related to the pathway leading to the onset of labor at term [26]. Elevated concentrations of HMG-1, considered the prototypic alarmin [47], have been demonstrated in cases of preterm delivery with intra-amniotic inflammation [48, 49] and in clinical chorioamnionitis at term [50]. We evaluated a few of the classical DAMPs such as HMG-1, HSP70, S100A8, and thrombospondin-1, and three of them (HMG-1, S100A8, and thrombospondin-1) were associated with shorter gestational duration. However, we did not find significant alterations in the concentrations of any of the examined cytokines. We theorize that the increase of DAMPs in early gestation is reflective of trimester-specific, localized events such as the fusion of the fetal membranes and establishment of the amniotic cavity [51] or growth and remodeling of the feto-placental unit, rather than an acute inflammatory process. Based on this, we further hypothesize that there is a relationship between the development of the uterine cavity and gestational duration. It should, however, be emphasized that our results are solely based on protein concentrations from amniotic fluid samples collected between 14 and 19 gestational weeks. These associations may not consist beyond this point.

In women with a spontaneous onset of labor at term, increased concentrations of MIP-1β, S100A8, and thrombospondin-1 were significantly associated with a decrease in gestational duration. MIP-1β has previously been demonstrated in cases of spontaneous PTD at < 34 weeks [52], as well as in women with symptoms of preterm labor who delivered within 7 days [6]. An upregulation of S100A8 is associated with chorioamnionitis/deciduitis [53]. S100-alarmins have also been described as essential immunoregulators in newborns, preventing excessive inflammation [54]. S100A8/A9 have been analyzed in breast milk; concentrations were significantly higher after term delivery, compared with PTD, and after vaginal delivery, compared with cesarean section [55]. Thrombospondin-1 is expressed in the placenta and has previously been reported in cases of small for gestational age (SGA) pregnancies and preeclampsia [56, 57].

Understanding the physiological events preceding parturition in healthy term pregnancies is essential in order to understand pathological pregnancies and, ultimately, possible prevention strategies. Aghaeepour et al. [14, 15] suggest the presence of a proteomic and an immune clock in women delivering at term, where deviations from precisely timed and chronological changes could potentially assist in the early prediction of adverse outcomes. However, they analyzed maternal sera and peripheral blood, repeatedly collected throughout gestation, while this study analyzed the protein composition of amniotic fluid collected at mid-trimester. The association between protein concentrations and gestational duration in this study though evokes the additional concept of a pregnancy clock that may also comprise the amniotic fluid. While the sample size was small, the lack of associations in the preterm group suggests that the immunological response in women with a spontaneous PTD may diverge from that of women delivering at term. However, further research is needed to confirm this.

The strengths of this study were particularly the robust methodology, including meticulous selection criteria, the extensive panel of selected candidate proteins, and the analysis of gestational duration as a continuous rather than a dichotomous (preterm/term) variable. We used two different statistical models and were able to show that the associations are robust to modeling choices. While the Cox regression is natural for modeling the survival outcome, a linear model can be more suited for detecting markers that have time-varying effects—this was previously shown to be likely based on Swedish birth demographics [58].

Furthermore, the cohort is unique due to its size, with a total loss-to-follow-up/missing information rate of only 0.97%. Another strength is that the results have been adjusted for covariates and experimental factors (plates); the latter are frequently neglected. Finally, we used a technology that offers a broader dynamic range and better accuracy, with lower inter- and intra-assay variation [59, 60], than other immunoassay technologies such as LUMINEX. To the best of our knowledge, this is the first study using a Meso-Scale Discovery approach with a broad panel of inflammatory markers in this context.

One limitation is that the study population of women undergoing invasive genetic testing is of advanced age with high risk or a history of chromosomal abnormalities. These circumstances, therefore, might not reflect the general low-risk pregnant population, limiting generalizability. The clinical translation value of our findings is also limited, and the results should rather be seen as interesting biological associations that may serve as a basis for future research. The results have been adjusted for plate effects, but other analytical conditions might have influenced the results. Researchers must be aware of the limitations of new markers and techniques during this rapidly expanding and developing era of biomarker research [60, 61].

## Conclusion

Mid-trimester amniotic fluid concentrations of thrombospondin-1, MIP-1β, and S100A8 were significantly associated with gestational duration at term, but not at preterm. This adds to the concept of a pregnancy clock, where our findings suggest that such a clock is also reflected in mid-trimester amniotic fluid. Further research is though needed to explore this. It is important to adjust for gestational age at sampling when performing amniotic fluid biomarker studies.

## Data Availability

All relevant data are within the manuscript.
